# Using mobile health technology to improve behavioral skill implementation through homework in evidence-based parenting intervention for disruptive behavior disorders in youth: study protocol for intervention development and evaluation

**DOI:** 10.1186/s40814-016-0097-4

**Published:** 2016-09-20

**Authors:** Anil Chacko, Andrew Isham, Andrew F. Cleek, Mary M. McKay

**Affiliations:** 1 Department of Applied Psychology, New York University, 246 Greene Street, New York, NY 10003 USA; 2 University of Wisconsin, 1513 University Ave, Madison, WI 53706 USA; 3 Silver School of Social Work, McSilver Institute, New York University, 1 Washington Square North, New York, NY 10003 USA

**Keywords:** Mobile health technology, mHealth, Children’s mental health, Homework, Disruptive behavior, Parenting intervention

## Abstract

**Background:**

Disruptive behavior disorders (DBDs) (oppositional defiant disorder (ODD) and conduct disorder (CD)) are prevalent, costly, and oftentimes chronic psychiatric disorders of childhood. Evidence-based interventions that focus on assisting parents to utilize effective skills to modify children’s problematic behaviors are first-line interventions for the treatment of DBDs. Although efficacious, the effects of these interventions are often attenuated by poor implementation of the skills learned during treatment by parents, often referred to as between-session homework. The multiple family group (MFG) model is an evidence-based, skills-based intervention model for the treatment of DBDs in school-age youth residing in urban, socio-economically disadvantaged communities. While data suggest benefits of MFG on DBD behaviors, similar to other skill-based interventions, the effects of MFG are mitigated by the poor homework implementation, despite considerable efforts to support parents in homework implementation. This paper focuses on the study protocol for the development and preliminary evaluation of a theory-based, smartphone mobile health (mHealth) application (My MFG) to support homework implementation by parents participating in MFG.

**Methods/design:**

This paper describes a study design proposal that begins with a theoretical model, uses iterative design processes to develop My MFG to support homework implementation in MFG through a series of pilot studies, and a small-scale pilot randomised controlled trial to determine if the intervention can demonstrate change (preliminary efficacy) of My MFG in outpatient mental health settings in socioeconomically disadvantaged communities.

**Discussion:**

This preliminary study aims to understand the implementation of mHealth methods to improve the effectiveness of evidence-based interventions in routine outpatient mental health care settings for youth with disruptive behavior and their families. Developing methods to augment the benefits of evidence-based interventions, such as MFG, where homework implementation is an essential mediator of treatment benefits is critical to full adoption/implementation of these intervention in routine practice settings and maximizing benefits for youth with DBDs and their families.

**Trial registration:**

ClinicalTrials.gov NCT01917838

## Background

This paper details the development and preliminary evaluation of My MFG, a mobile health application, to support parents participating in the multiple family group (MFG) intervention for the treatment of disruptive behavior disorders (DBs) (i.e., oppositional defiant disorder (ODD) and conduct disorder (CD)) in children. My MFG was developed to improve the implementation of behavioral parenting skills learned during MFG groups in order to maximize the potential benefits of MFG. This paper discusses the rationale behind the development of My MFG, the conceptual models that form the foundation of My MFG, and a proposal for a series of iterative studies to develop and preliminarily evaluate My MFG.

### Evidence-based treatment for DBDs in socioeconomically disadvantaged settings

Publicly funded outpatient mental health settings are one of the main contexts where youth with DBDs receive mental health services in socioeconomically disadvantaged communities. Evidence-based treatments, primarily those that focus on supporting caregivers (oftentimes parents) in implementing skills to modify child behavior, have been developed for treating DBDs [1]. Studies have shown that these skill-based parenting interventions can improve outcomes for youth affected with DBDs, even those from higher risk backgrounds [2–7].

A central aspect of response to most of these evidence-based parenting skills interventions is homework, which is between-session exercises where the client (i.e., parent) practices specific skills learned within-session in order to promote skill acquisition [8, 9]. Although limited, studies of evidence-based parenting interventions suggest that homework completion is related to improved outcomes, even after taking into account parent/child characteristics (baseline severity of mental health problems, motivation), attendance at treatment sessions, and within-session participation [10–17]. Collectively, data suggests that attending treatment and being actively involved when in a treatment session is necessary, but insufficient in producing desired outcomes following treatment—homework completion is necessary to maximize benefits of evidence-based parenting interventions [8, 11, 13, 15]. Unfortunately, the limited data available suggest that homework is not often completed in parenting interventions [2, 18]. As such, attention to how best to support parents in completing homework is also needed in order to attain the full potential therapeutic benefits of parenting interventions as well as to maximize the chances that these interventions will be fully adopted/implemented in clinical settings.

### Multiple family groups for DBDs

The multiple family group (MFG) intervention is an evidence-based parenting intervention for the treatment of youth with DBDs who present to outpatient mental health clinics in socioeconomically disadvantaged communities [19, 20]. As a foundation, MFG focuses on common, effective practices across evidence-based parenting interventions for treating DBDs [21, 22] represented as the “4Rs” (i.e., rules; responsibility; relationships; respectful communication) and factors related to family engagement in mental health services, represented as “2Ss” (stress and social support). These empirical literatures are translated into core skills, processes, and methods and are delivered in a manual-guided, flexible manner. MFG uses a multi-family group delivery model to increase engagement in services and provide an effective and efficient service-delivery mechanism. In fact, data from a large scale effectiveness study of MFG in outpatient mental clinics in urban and socioeconomically disadvantaged communities found that MFG resulted in significant benefits relative to services-as-usual at immediate post-treatment and follow-up assessments on DBD outcomes [19, 20].

Importantly, higher levels of homework completion during MFG results in considerably greater effects on child DBD symptoms [19, 20]. As such, skill implementation through homework is an important process for attaining maximal benefits from MFG. Within MFG, homework implementation has been supported through use of multiple empirically supported strategies (e.g., motivational techniques, problem solving barriers to skill implementation, and phone calls between session, as well as home visits [10, 19]). However, despite these significant efforts, rates of homework completion were often poor [19, 20]. The lack of significant benefits of evidence-based approaches used to support the homework implementation process for families engaged in MFG points to a clear need for alternate methods to address this critical issue that attenuates benefits of this intervention approach. Similarly, the considerable efforts that are typically taken to ensure homework implementation poses significant barriers for clinicians in adoption/implementation of MFG. Collectively, greater attention to supporting the homework process has both the potential to enhance clinical effectiveness of evidence-based interventions and to promote greater adoption/implementation of these interventions in clinical practice settings.

### The homework process

Kazantzis and colleagues have pioneered a framework that integrates well-established models of behavior change into an integrated social-cognitive-behavioral theory of the homework implementation process [9]. This model proposes four processes involved in homework implementation within the context of psychotherapy: (1) designing; (2) assigning; (3) doing; and (4) reviewing homework. The DADR model posits that specific social, cognitive, and behavioral factors related to the homework task (e.g., perceived difficulty and relevance of the task), provider (e.g., ability to tailor the homework task; address barriers and support facilitators to completion; reinforce homework implementation attempts), client (e.g., perceived self-efficacy; motivation; realized difficulty in implementing homework tasks; costs, benefits, and relevance of the assignment; severity of mental health problems and stress), and environment (e.g., social support to implement homework; environmental barriers and facilitators to homework completion) affect the quality of each of the four processes, ultimately impacting the quantity and quality of homework implementation [23, 24].

Data from surveys of treatment providers, evidence-based treatment developers, and randomized clinical trials offer limited but useful information regarding the homework implementation process within the context of evidence-based treatments, including skill-based parenting interventions, and suggest that despite the common use of homework in evidence-based treatments for youth and families, the process of designing homework tasks is rarely theory-driven or investigated [25–28]. Methods to support homework implementation must attend to designing, assigning, and reviewing homework within-session to support successful implementation of homework between-sessions (do process). Unfortunately, methods to more effectively design homework have been limited and typically focused on varying the complexity (frequency or intensity) of the homework task. Motivation to try a new behavior is generally assumed or addressed in the assign phase rather than designing homework to be motivating itself. In addition, homework is assigned in a confined environment but carried out in a natural environment where the aforementioned barriers to successful practice are likely to exist. A homework task may be simplified in order to make it easier to adopt, but may then be too small a change to be successful in a real world environment. Moreover, methods to address the homework at the time and place that it is completed are limited and/or unrealistic for wide-spread use in routine clinical care. Use of reminders as cues to perform the homework task in context has been routinely used but is often limited in improving completion [8]. The use of telephone-contacts or home-visits to support homework implementation is more effective but is time-intensive and often not feasible within the context of routine outpatient mental health services [19]. As such, development of more cost-efficient, theory-driven methods to design homework to maximize motivation and learning in context and support effective recall and implementation of homework in context is an important goal.

### Mobile health applications: potential to improve the homework process

Mobile health (mHealth) offers a practical and effective delivery mechanism to address many of the difficulties with the homework process. mHealth, facilitated through smartphones, has offered clients 24/7 contact with information, support, and clinical expertise. mHealth has been increasingly utilized in various health fields given high levels of ownership of smartphones, portability of smartphones; allowing for constant access; flexibility of software to quickly be modified for use with different populations, and potential for integration of data within electronic health records. mHealth offers the ideal mechanism for addressing the homework process. In particular, mHealth can impact how homework tasks are developed and how these homework tasks are implemented (design and do process). Through utilizing the flexible technology and features of smartphones, homework tasks can be designed to support and enhance factors known to affect the design process (e.g., perceived self-efficacy and motivation) as well as the do process (e.g., improving social support and effective recall of homework). There is a lack of research that addresses the use of mobile technology to improve homework completion and quality and often a lack of theoretical foundation supporting mobile technologies in behavioral interventions [29, 30].

### Overview of study

Evaluating the effectiveness of a conceptual model for improvement of treatment via tools that support skill implementation in an uncontrolled (home and community) environment requires an iterative design model to ensure the tools work in the environment in which they are designed to be effective. This process requires multiple prototypes and feedback from potential end users to ensure the design delivers the content effectively and efficiently. Effective design means that it provides an appropriate response in an engaging manner. Efficient design is easy to use and works even in low signal areas. We describe below a process of an effective design/development/pilot testing process and how this process will be utilized to evaluate My MFG and the study protocol within a short time-frame. Our study is divided into three distinct, iterative phases. Phase I: pilot study I will be aimed at the initial development of the My MFG application with a small open clinical trial. The aim of this phase will be to determine the palatability, feasibility, and technical issues of the My MFG application for both consumers and MFG facilitators, develop efficient and effective methods for training MFG facilitators on the use of the My MFG application, and to refine the intervention. Phase I: pilot study II will be an evaluation of the revised My MFG application. The aim of this phase will be to determine the palatability, feasibility, and technical issues of the revised My MFG application for both consumers and MFG facilitators, and further develop efficient and effective methods for training MFG facilitators on the use of the My MFG application. Further revisions will be made for the final My MFG application. Phase II: small-scale pilot randomised controlled trial will focus on gathering preliminary data on a My MFG usage, improvements in the homework process (quantity and quality of homework) during MFG, satisfaction with the My MFG application, and determining refinements of the study protocol for use in a future large-scale RCT. Secondary aims will include a comparison of MFG plus My MFG compared to MFG-alone on various homework process outcomes. We describe these two-phase, three study designs below. Importantly, all participating families in the studies will utilize their own smartphones or are provided a smartphone for the duration of the project.

## Methods/design

### Phase I: developing My MFG

#### Phase I: pilot study I

To build a tool that will be used as desired, the study will be designed to include close collaboration with potential end-users of the program (i.e., MFG clinicians and families who have participated in MFG in previous studies). Initial discussions with staff and a focus group of prior patients will identify program-specific key processes and supports needed and the times or situations in which they are needed. These initial discussions will lead to a preliminary design whose elements can be shared with the MFG clinicians and prior patients for feedback on the initial concepts. Once the conceptual design is agreed upon, a prototype of just the user interface will be developed for feedback and testing. The prototype user interface will identify how logical the icons and language are, whether the order of operations makes sense to the end-user and other important end-user issues. Because it is only the design of the user interface that is being tested, designs will be modified as end-users make recommendations and responses to the changes can be captured immediately. After user interface issues are resolved, a basic data infrastructure will be built to pilot test the application.

#### Designing My MFG

**Fig. 1:**
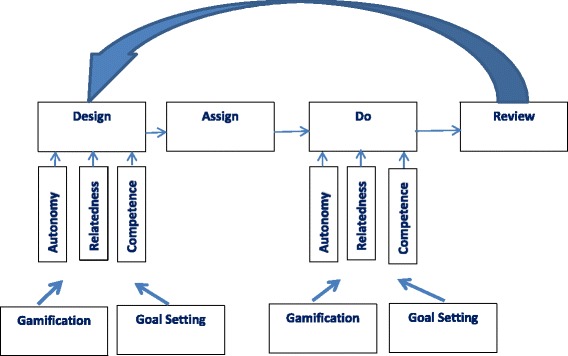
My MFG conceptual model

The design of My MFG is based on the overlap of four theoretical models (Fig. 1). The first is the DADR model of the homework process [9]. The second is self-determination theory [31] which posits that behavior change and learning are determined by three factors: autonomous motivation, relatedness and competence. The third is gamification [32] which aims to increase motivation by making tasks that are not inherently motivating fun and rewarding. The fourth is goal setting [33], which assists individuals in directing attention, arousal, discovery, and or/use of task relevant knowledge and strategies toward goal-setting activities and away from goal-irrelevant activities.

#### Key My MFG components

My MFG intervention components summarized below are aimed at addressing barriers (e.g., perceived efficacy; stress) and supporting facilitators (e.g., motivation; social support; effective recall; associated benefits) within the “design” and “do” processes of the DADR homework model through enhancing autonomous motivation, competence, and relatedness, which have been utilized in our previous studies that have relied upon self-determination theory as a guiding model [34, 35] As such, we will adapt these components for use in My MFG.

#### Tools to support the development of autonomous motivation

##### *Push elements*

Families enter data to trigger timely text and audio reminders of significant milestones, reasons for implementing homework, and inspirational messages.

##### *Gamification/goal setting*

My MFG family games focused on skill development will further promote active engagement and autonomous motivation, as will the personalized goal-setting procedure with rewards and recognition for meeting goals. My MFG will employ multiple visual displays and content and features will be updated frequently to provide the family an engaging evolving experience with the My MFG application.

#### Tools to develop competence

##### *Questions and answers*

“ Brief answers to existing frequently asked questions about child behavior and MFG such as “Ways to recall and use homework skills,” will be provided along with links to other My MFG services that offer more detailed support for parents. Parent designed “What do I do now?” FAQs will offer single button touch link to skill implementation support during difficult times. *Easing distress* will translate content on stress management currently utilized in MFG to My MFG.

##### *Triage and feedback*

Using information collected during setup and the weekly check-in with questions about family goals, use of MFG skills, levels of stress and social support and other risk or protective factors, My MFG will provide links to relevant My MFG resources. An example of what would happen if a participant is prompted and then accepts support: many parents experience stress at managing a child’s behavior at a specific time in the day (e.g., getting ready for bed). My MFG will remind (push-in) families at selected times (e.g., 30 min before bedtime) of what skills to use, offer MFG stress management exercises, or connect to online peer support from other members of the MFG group. Triage and feedback is intended to derail the automatic punitive and reactive family interactions and parenting discipline strategies often observed in families of youth with DBDs which maintain the disruptive problems experienced by the youth (and family) by providing the family with just-in-time, tailored coping supports and reminders to implement MFG homework skills when and wherever they may be needed. My MFG will help families focus on developing the 4Rs of MFG while goal setting will augment the quality of the homework implementation.

#### Social support services (relatedness)

##### *Discussion groups*

Participants can exchange emotional support and information with others assigned to their MFG group via online support groups. They can share their progress in achieving My MFG goals and ask questions of both group participants and facilitators.

All of the tools that will be included lay self-determination theory over the DADR model of homework implementation, providing tools that motivate and engage, improve competence, or provide social support for the client during the critical do process. Tools can be customized during the design phase of the homework process and modified during the review phase of the homework process during the MFG group. The game can be adaptive to families via simple initial tasks and leveling-up to more difficult tasks that are related to more difficult to adopt behaviors.

An initial pilot study with a small sample of six to eight families will test the MFG plus My MFG mHealth application for palatability, feasibility, and technical issues. This initial feasibility pilot will assist in identifying the best strategies for recruitment, enrollment, data collection, and technical aspects of delivering My MFG. Feedback from study participants will be systematically collected on an ongoing basis during MFG to rapidly revise, refine, and update My MFG components during the course of treatment while also finalizing My MFG for a second pilot with new participants. MFG clinician feedback will be used to determine barriers and facilitators to using My MFG and clinical and regulatory issues of integrating My MFG in practice. Specifically, the objectives of the pilot I will be:

1. Determine if initial prototype is palatable for consumers. Do consumers (i.e., families) find the content/methods in My MFG helpful in supporting homework? Are there aspects to homework support that is insufficiently addressed in My MFG? What new content/methods can be incorporated to further support homework? Do consumers like using My MFG?
2. Determine if the initial prototype is feasible for consumers. Do consumers (i.e., families) find My MFG easy to implement daily? What are the challenges to implementing My MFG consistently?
3. Determine if the initial prototype is technically sound for consumers. Do consumers (i.e., families) find My MFG easy to navigate? What are the challenges in programming My MFG for use? What are modifications, if any, to further improve navigation of My MFG? Do consumers find the steps to personalize My MFG easy to implement?
4. Determine if the initial prototype is palatable and feasible for MFG Facilitators. Do MFG Facilitators utilize the My MFG in assigning and designing homework in MFG groups? What are the challenges in utilizing My MFG in assigning and designing homework? Do MFG Facilitators utilize My MFG in reviewing homework in MFG groups? What are the challenges in utilizing My MFG in reviewing homework? What are the challenges in integrating My MFG into practice in their clinical setting? What modifications, if any would facilitators recommend in improving My MFG so it aligns better to MFG and better supports the homework process?
5. Determine the most efficient and effective manner to train and support MFG facilitators in learning and implementing My MFG into MFG groups. Does initial training meet the needs of facilitators in learning and implementing My MFG into MFG? What recommendations do facilitators have to improve training?

#### Phase I: pilot study II

A second pilot study of MFG plus the revised My MFG will then be conducted to gather information from a different user group (six to eight families) who had not experienced the transformation of the prototype application. Feedback from study participants as well as MFG clinicians will be collected on an ongoing manner during MFG to rapidly revise, refine, and provide updated My MFG components/processes during the course of treatment while also finalizing My MFG prior to conducting a phase II small-scale pilot randomized controlled trial. Specifically, the objectives of the pilot II will be to:

1. Determine if the revised prototype is palatable for consumers. Do consumers (i.e., families) find the content/methods in My MFG helpful in supporting homework? Are there aspects to homework support that is insufficiently addressed in My MFG? What new content/methods can be incorporated to further support homework? Do consumers like using My MFG?
2. Determine if the revised prototype is feasible for consumers. Do consumers (i.e., families) find My MFG easy to implement daily? What are the challenges to implementing My MFG consistently?
3. Determine if the revised prototype if technically sound for consumers. Do consumers (i.e., families) find My MFG easy to navigate? What are the challenges in programming My MFG for use? What are modifications, if any, to further improve navigation of My MFG? Do consumers find the steps to personalize My MFG easy to implement?
4. Determine if the revised prototype is palatable and feasible for MFG Facilitators. Do MFG facilitators utilize My MFG in assigning and designing homework in MFG groups? What are the challenges in utilizing My MFG in assigning and designing homework? Do MFG Facilitators utilize My MFG in reviewing homework in MFG groups? What are the challenges in utilizing My MFG in reviewing homework? What are the challenges in integrating My MFG into practice in their clinical setting? What modifications, if any would facilitators recommend in improving My MFG so it aligns better to MFG and better supports the homework process?
5. Determine the most efficient and effective manner to train and support MFG facilitators in learning and implementing My MFG into MFG groups. Does the revised training meet the needs of facilitators in learning and implementing My MFG into MFG? What recommendations do facilitators have to improve training?

#### Analysis of phase I: pilot study I and pilot study II

Quantitative assessments and qualitative focus groups will be completed by consumers of My MFG after each MFG treatment session during the pilot studies to assess for palatability, feasibility, and technical issues. Likert-scale measures will be developed to allow consumers to rate aspects of each My MFG component for issues of palatability, feasibility, and technical issues. Scores below predefined cutoffs on these assessments, indicating concerns/problems, will be identified and used to refine the My MFG application. These scores will also serve as discussion points for the weekly post-session focus groups. Additionally, the weekly post-session focus groups will allow for consumers to provide feedback on all aspects of the My MFG application. The combination of weekly post-session assessments by consumers and weekly focus group discussions will allow for ongoing feedback on specific issues of palatability, feasibility, and technical issues while also allowing consumers to provide open feedback to further improve My MFG to meet consumer’s needs.

### Phase II: small-scale pilot randomised controlled trial

A small-scale pilot randomised controlled trial will be conducted to further understand feasibility, utility, and palatability, determine whether a “signal” can be detected, and to evaluate/refine the study protocol.

### Setting and recruitment

Participating outpatient mental health clinics in the New York City area will serve as sites for this study. Providers at the participating clinics will receive information about the proposed study and will have printed materials to provide to their clients about participation in the proposed study. Potentially eligible youth and their families (based on an intake diagnosis of ODD and CD made by clinical service providers) will be informed of the study by their providers (step I) and then, if the family is interested in learning more about the study, contacted by a member of the research staff (step II). In phase II, informed consent/assent will be completed by the family, which provides study details as well as specify the possibility of being selected for the MFG-alone or MFG plus MY MFG conditions via random assignment. If the youth and family is screened as eligible, and consent/assent is given, then study staff will contact the project director (blind to family information) who will assign the family to one of the two study conditions based on predetermined sequence based on random permutation calculation.

#### Inclusion criteria

Families will be included if they meet the following criteria: (1) youth between the ages of 7 to 13 years and an accompanying adult primary caregiver available to participate in the research and intervention activities; (2) English-speaking youth and adult caregiver; and (3) youth meeting criteria for DBD via standardized assessment procedure.

#### Exclusion criteria

Children will be excluded if there is evidence of psychosis. In addition, if the youth or adult caregiver presents with emergency psychiatric needs that require services beyond that which can be managed within an outpatient setting (e.g., hospitalization, specialized placement outside the home), active intervention by clinic and research staff to secure what is needed will be made. Children will not be excluded if they participate in other psychosocial or pharmacological interventions.

#### Treatment assignment and sample size rationale

**Fig. 2:**
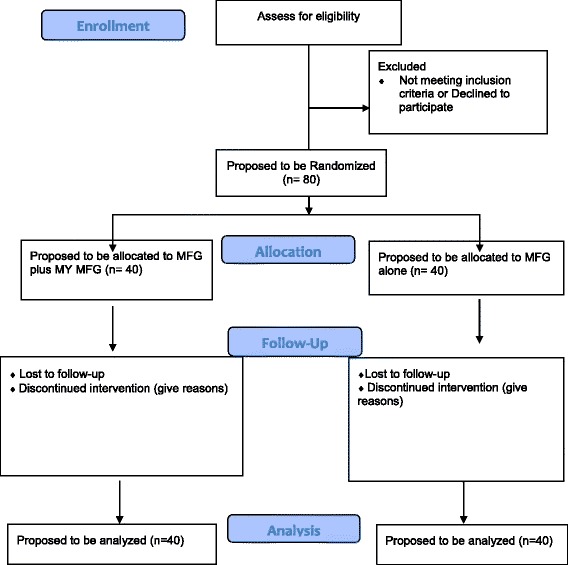
CONSORT flowchart

Families will be assigned on a 1:1 basis to MFG alone (*n* = 40) or MFG plus My MFG (*n* = 40) (see Fig. 2 CONSORT flowchart) via predetermined sequence based on random permutation calculation by the project director, who is blind to family information. Both treatment conditions will run in parallel within each participating clinic. Sample size will be determined based on previous randomized controlled trial data suggesting that a sample size of 40 participants per condition would provide a meaningful difference in homework completion between those that are typically highly engaged and those that are less engaged.

### Interventions

#### Multiple family group

Multiple family group (MFG) is a 16-week service delivery strategy that was guided by a manualized protocol. Each group included six to eight families, composed of identified youth, their adult caregiver(s), and sibling(s) between the ages of 6 and 18. As a foundation, MFG takes a common elements approach by identifying essential components from the empirical literature regarding core effective practices for treating DBDs, represented as the 4Rs (i.e., rules; responsibility; relationships; respectful communication) and factors related to family engagement in mental health services, represented as 2Ss (stress and social support. Each of the 16 sessions that focused on one of the 4Rs and 2Ss and proceeded with the following processes: (a) creating social networks; (b) information exchange/homework review; (c) group discussion regarding the skill; (d) individual family practice; and (e) homework assignment.

#### Multiple family group plus MY MFG

MFG plus MY MFG treatment condition has the same format, content, processes, and methods as MFG except that the homework process (homework review/homework assignment) is conducted through the My MFG application.

#### Measures

Data will be collected at baseline, pretreatment, at each session, and at post-treatment. See Table 1 for the data collection tools and timing of the collection of each measure. My MFG usage data (e.g., frequency, duration of use, which components used, etc.) will also be collected directly from servers throughout treatment.

**Table 1:** Outcome measures for pilot randomised controlled trial phase

Domain	Measure	Reporter	Time of assessment
Homework process; homework quantity and quality	Homework rating scale-II [9]	Parent and clinician	Every session
Homework process	Homework adherence and competence scales [9]	Independent observer	Every session
Attendance	Session attendance	Clinician	Every session
Consumer satisfaction and feedback	Treatment attitude inventory [40] Feedback questionnaire Group and individual consultancy meeting	Parent; target child; therapist	Postreatment

#### Primary and secondary aims

The primary aim will be to obtain summary statistics of the key outcomes (quality of the design, do, and review phases, quality of homework completed), to determine usage of the My MFG application, and to identify challenges and barriers for uptake of My MFG application. An additional primary aim will be to determine the feasibility of the study protocol. Secondary analyses will compare MFG-alone to MFG plus My MFG on key homework process outcomes. The following hypothesis for this phase includes

1. Greater quality of the “design” and “do” process rated by clinicians, parents, and independent coders. As My MFG is being developed to specifically address the design and do process, we hypothesize that clinicians, parents, and independent coders will rate the quality of these two processes to be high. We also hypothesize that MFG plus My MFG, compared to MFG-alone, will result in significantly better design and do process outcomes.
2. Greater quantity and quality of homework assignments rated by clinicians and parents. Given aim #1 and hypothesis #1, we hypothesize that the quantity and quality of homework completed will be high for the MFG plus My MFG group. We also hypothesize that MFG plus My MFG, compared to MFG-alone, will result in significantly better scores on homework adherence and quality measures.
3. Greater quality of the “review” process as rated by clinicians, parents, and independent coders. Given aim #2 and hypothesis # 2, we hypothesize that the review process during the MFG plus My MFG condition will be rated high by families (based on improved adherence to and quality of homework completed) as review of homework requires that homework be completed in a high-quality manner. Low rates and quality of homework limits opportunities for effective review. We also hypothesize that MFG plus My MFG, compared to MFG-alone, will result in significantly better scores on the review process.
4. Greater satisfaction with treatment as rated by the parent, target child, and clinicians. We hypothesize that families and clinicians in the MFG plus My MFG condition will have high levels of satisfaction with the treatment process, given that the goal of My MFG is to enhance autonomous motivation, competency, and relatedness while enhancing the process of homework during MFG. We also hypothesize that MFG plus My MFG, compared to MFG-alone, will result in significantly higher satisfaction with treatment.

Additional primary aim: An additional primary aim will be to determine the feasibility of conducting the RCT protocol. Specific aims include whether the study protocol will allow for:

1. Recruitment of 80 participants
2. Retention of 80 % of the sample
3. Efficient training of MFG facilitators
4. Efficient data collection

#### Data analyses

Given that the pilot RCT is underpowered to detect differences between groups, our primary analyses will focus on descriptive summary statistics (e.g., means and standard deviation with confidence intervals). Specifically, summary statistics will be compared to established cutoffs on measures. This approach will allow for direct comparison of study summary statistics to established summaries allowing for assessment of various aspects of the DADR homework process (i.e., design, do, and review phases, quality of homework completed). Summary statistics of quantity of homework completed will be directly compared to our previous studies of MFG [9]. Moreover, completion of the measures every session will also allow for determining how each component of the My MFG application is affecting the homework process over time and for identifying key content and processes that may require further development. Collectively, this primary data analytic plan will determine the extent to which the proposed My MFG application affects key aspects of the homework process and allows for gathering of information to further refine the My MFG application.

We will gather information through the feedback questionnaire as well as post-treatment group and individual collaborative consultancy meetings regarding My MFG content, (parents, children, and clinicians) in order to further refine My MFG. We will also utilize My MFG usage data via servers as probes to obtain further information from participants regarding My MFG. The data from these sources will allow us to further understanding patterns of use (e.g., identify patterns of low usage over treatment; identify underutilized content, etc.), consumer challenges (processes, facilitators, and barriers to uptake from participants in using the My MFG application), and allow for further suggestions for refining the My MFG application.

We will also analyze feasibility and effectiveness of the study protocol through ongoing feedback from research staff and MFG facilitators. Information gathered from MFG facilitator/research staff will assist in understanding barriers to implementing the RCT study protocol (i.e., recruitment, retention, facilitator training, and data collection).

Secondary analyses will focus on between group (group: MFG plus My MFG and MFG-alone) X 2 (time: pre-treatment and post-treatment) repeated measures multivariate analysis of variance (MANOVA) to address homework quantity and quality at each session, average homework quantity and quality across all sessions, and quality of the design, do, and review processes. Alpha-inflation correction procedures will not be used for subsequent contrasts given the exploratory nature of the study. Follow-up analyses will include calculation of effect size by subtracting the MFG plus My MFG group mean from the MFG-alone group mean and dividing by the pooled standard deviation of the groups. Effect size data will be an important indicator of a “signal” that My MFG may be improving the homework process given the relatively small sample size of this study. Independent sample *t* tests will be used for determining differences between treatment conditions on consumer satisfaction to treatment. It is important to state that the results of these secondary analyses will be treated with caution given the small sample size of the RCT.

##### Current status of the study

Phase I pilot studies have been completed with further refinement to My MFG application being made and refinements to the study protocol. Phase II: small-scale pilot randomised controlled trial was recently completed and study data are being coded, cleaned, and analyzed.

## Discussion

The National Institute of Mental Health [36, 37] in the United States has specifically called for acceleration of research to maximize the potential of current treatments to reduce symptoms and enhance functioning while improving quality of and lowering the cost of care. The mHealth application and methods proposed herein serve as systematic, theory-driven approach to significantly advance understanding of how best to support the homework process—a common element of many evidence-based treatments across various disorders and populations [25]. Ultimately developing methods to support homework implementation should result in greater implementation and generalization of behavioral skills learned during treatment which should maximize the effectiveness of these evidence-based treatments.

Given the prevalence of DBDs and the limited resources available in outpatient mental health clinics serving disadvantaged communities, maximizing the potential effectiveness and efficiency of existing evidence-based treatments for DBDs, such as MFG, is a high public health priority. We hypothesize that My MFG, which is based on a strong theoretical foundations [9, 31–33], employed through mHealth features via smartphones we have successfully utilized in previous work [34, 35] offers an opportunity to significantly improve the effectiveness of MFG. Importantly, the data gathered through the series of pilot studies described herein allow for a preliminary evaluation and development of the My MFG application. Summary statistics data from the RCT study will inform the extent to which the My MFG application results in high levels of quality and quantity in the homework process and allows for identification of specific content, processes, and/or methods employed in My MFG application that do not appear to result in high rates of quality homework process. The secondary analyses, while underpowered, will allow for detection of a possible “signal” that suggests benefit of My MFG relative to MFG alone. Qualitative feedback from consumers and user data from the My MFG application will augment the session-by-session data. Collectively, the multi-method and multi-informant strategy will allow for further refinement of the my MFG application for potential evaluation in a well-powered subsequent RCT. Lastly, the study RCT will allow us to gauge an important aim—the extent to which the study protocol is feasible and whether and what modifications will need to be made prior to a larger scale evaluation.

Utilizing relatively simple mHealth methods to augment the benefits of existing psychosocial treatments should also improve the quality of overall care for families while not significantly increasing the burden of treatment for both families and clinicians. There is significant data demonstrating that the demands of parenting interventions, in terms of amount of time, effort, and resources needed from families to participate in the intervention, are directly related to engagement and dropout from treatment [38]. mHealth offers the opportunity to streamline parenting interventions to be less demanding upon families and thereby increasing the chances that interventions can be more readily implemented, particularly in mental health settings serving families from resource-poor communities. Likewise, interventions can be burdensome for clinicians, resulting in difficulties with overall implementation and sustained use [19, 39]. mHealth methods may offer the promise of replacing effective but time-intensive approaches to supporting homework (e.g., phone call reminders), thereby decreasing the burden of an intervention for clinicians. Ultimately, mHealth methods may prove to be vital to augment evidence-based interventions and increase the chances of successful adoption/implementation of these interventions in clinical practice settings.

## Abbreviations

CD: Conduct disorder
DBDs: Disruptive behavior disorders
MFG: Multiple family group
mHealth: Mobile health
ODD: Oppositional defiant disorder
